# A standardized European hexagon gridded dataset based on OpenStreetMap POIs

**DOI:** 10.1016/j.dib.2023.109315

**Published:** 2023-06-14

**Authors:** Dakota Aaron McCarty, Hyun Woo Kim

**Affiliations:** a Department of Urban Policy & Administration, Incheon National University, 119 Academy-ro, Yeonsu-gu, Incheon 22012, South Korea; b Urban Science Institute, Incheon National University, 119 Academy-ro, Yeonsu-gu, Incheon 22012, South Korea

**Keywords:** Point of interest, Urban planning, Amenities, Services, Hexagon grid, TF-IDF

## Abstract

Point of interest (POI) data refers to information about the location and type of amenities, services, and attractions within a geographic area. This data is used in urban studies research to better understand the dynamics of a city, assess community needs, and identify opportunities for economic growth and development. POI data is beneficial because it provides a detailed picture of the resources available in a given area, which can inform policy decisions and improve the quality of life for residents. This paper presents a large-scale, standardized POI dataset from OpenStreetMap (OSM) for the European continent. The dataset's standardization and gridding make it more efficient for advanced modeling, reducing 7,218,304 data points to 988,575 without significant resolution loss, suitable for a broader range of models with lower computational demands. The resulting dataset can be used to conduct advanced analyses, examine POI spatial distributions, conduct comparative regional studies, and research to help enhance the understanding of the distribution of economic activity and attractions, and subsequently help in the understanding of the economic health, growth potential, and cultural opportunities of an area. The paper describes the materials and methods used in generating the dataset, including OSM data retrieval, processing, standardization, hexagonal grid generation, and point count aggregations. The dataset can be used independently or integrated with other relevant datasets for more comprehensive spatial distribution studies in future research.

**Specifications Table**

**Table:** 

Subject	Planning and Development
Specific subject area	Large-scale point-of-interest (POI) extraction, processing, optimization, and mapping across the European continent
Type of data	Table (GeoPackage, .gpkg) QGIS project (.qgz)
How the data were acquired	Python script was used to download and process OpenStreetMap (OSM) data (extracted from GeoFabrik).
Data format	Raw Analyzed
Description of data collection	GeoFabrik data (retrieved April 20, 2023) was processed to extract point-of-interest data, points with a *shop* or *amenity* tag. Next, the data points were standardized using TF-IDF and manual processing to align with OSM tag/key documentation. Then, category labels based on documentation review were added. Finally, the dataset was spatially joined and aggregated to hexagonal grids in the study area to obtain POI counts by category, using four spatial resolutions: hex5 (∼250km**^2^**), hex6 (∼35km**^2^**), hex7 (∼5km**^2^**), and hex8 (∼0.7km**^2^**).
Data source location	Based on OpenStreetMap and GeoFabrik's Europe region, being Albania, Andorra, Austria, Azores, Belarus, Belgium, Bosnia-Herzegovina, Bulgaria, Croatia, Cyprus, Czech Republic, Denmark, Estonia, Faroe Islands, Finland, France, Georgia, Germany, Great Britain, Greece, Guernsey-jersey, Hungary, Iceland, Ireland and Northern Ireland, Isle of Man, Italy, Kosovo, Latvia, Liechtenstein, Lithuania, Luxembourg, Macedonia, Malta, Moldova, Monaco, Montenegro, Netherlands, Norway, Poland, Portugal, Romania, Russia, Serbia, Slovakia, Slovenia, Spain, Sweden, Switzerland, Turkey, and Ukraine.
Data accessibility	All relevant data and Python script were deposited on: Repository name: Zenodo Data identification number: 7885644 Direct URL to data: https://doi.org/10.5281/zenodo.7885644

## Value of the Data

• This standardized Point of Interest (POI) dataset offers valuable insights into various areas, such as mobility, culture, nightlife, community, economic activity, and public services across a study region.
• Hexagonal grid sampling provides a consistent, accurate distribution across the study area, creating a flexible foundation for versatile exploration and data analysis for researchers.
• By employing standardization and gridding, this dataset becomes more efficient for advanced modeling, reducing 7,218,304 data points to 988,575 without significant resolution loss (retaining ∼0.7km^2^), making it suitable for a broader range of models with lower computational demands.
• Using the already standardized terms from the OSM documentation, standardization of the data was possible through the Term Frequency - Inverse Document Frequency (TF-IDF) algorithm. This methodology is widely applicable to problems with misclassification, reclassification, and text misalignment.
• Researchers can leverage this data to conduct advanced analyses to examine POI spatial distributions and conduct comparative regional studies, enhancing understanding of the economic activity, distribution, attractions, and, subsequently, economic health, growth potential, and cultural opportunities.
• This dataset can be utilized independently or integrated with other relevant datasets, such as demographics or road networks, for more comprehensive spatial distribution studies in future research.

## 1 Objective

OpenStreetMap (OSM) provides a range of openly available urban datasets [1]. However, as it is a community-driven effort, standardization is frequently lacking, making the dataset hard to use at scale or in comparative analyses. Additionally, with large datasets come additional limitations with processability due to computing limits. This dataset aims to address both problems through obtaining highly valuable point-of-interest (POI) data [2]; and through careful standardization of the POI data and further spatial point count aggregation into a hexagonal grid. The hexagonal grid was selected over other types of grids as research has demonstrated its ability to preserve spatial clustering patterns while also providing equal emphasis to every areal unit in visualizations due to its equidistant nature [3,8]. There is an additional benefit to using hexagonal grids when considering future studies, as they offer significant advantages in examining cell connectivity, particularly in conveying levels of connectivity within and among areas compared to other available grids [9]. As a result, the final dataset is more user-friendly and optimized, being more readily usable with a broader range of algorithms, software, and studies.

Researchers have highlighted the potential of OSM POI data to drive urban science research and study urban change, despite its imperfections [4]. Barrington-Leigh & Millard-Ball [5] assessed the completeness of OSM road data globally, finding that in many places, researchers and policymakers can rely on the completeness of OSM or will soon be able to do so. This limitation of completeness is a strong driver of our research, where we are attempting to better standardize and fill in the gaps within the OSM POI datasets. A third study [6] analyzed pedestrian streets in 992 cities around the world using OSM data and spatial analysis techniques, revealing a chasm in car-free development mainly between Southern and Western European cities and their peers in other continents. Another study explored OSM data, highlighting its use in simplifying and visualizing complex urban data from street networks and buildings worldwide. It emphasized that the prevalence of urban data and computational power can lead to new, comprehensive analyses of urban forms from both qualitative and quantitative perspectives [7]. Together, these studies demonstrate the value of crowdsourced geographic databases like OSM for spatial data collection and urban planning, as well as the need for ongoing quality control measures. This study attempts to help fill the gaps in these control measures through the developed methodology to provide a more standardized and complete OSM POI dataset.

## 2 Data Description

The dataset has been collected from GeoFabrik based on their “Europe region,” comprised of 50 subregions: Albania, Andorra, Austria, Azores, Belarus, Belgium, Bosnia-Herzegovina, Bulgaria, Croatia, Cyprus, Czech Republic, Denmark, Estonia, Faroe Islands, Finland, France, Georgia, Germany, Great Britain, Greece, Guernsey-jersey, Hungary, Iceland, Ireland and Northern Ireland, Isle of Man, Italy, Kosovo, Latvia, Liechtenstein, Lithuania, Luxembourg, Macedonia, Malta, Moldova, Monaco, Montenegro, Netherlands, Norway, Poland, Portugal, Romania, Russia, Serbia, Slovakia, Slovenia, Spain, Sweden, Switzerland, Turkey, and Ukraine.

**Fig. 1:**
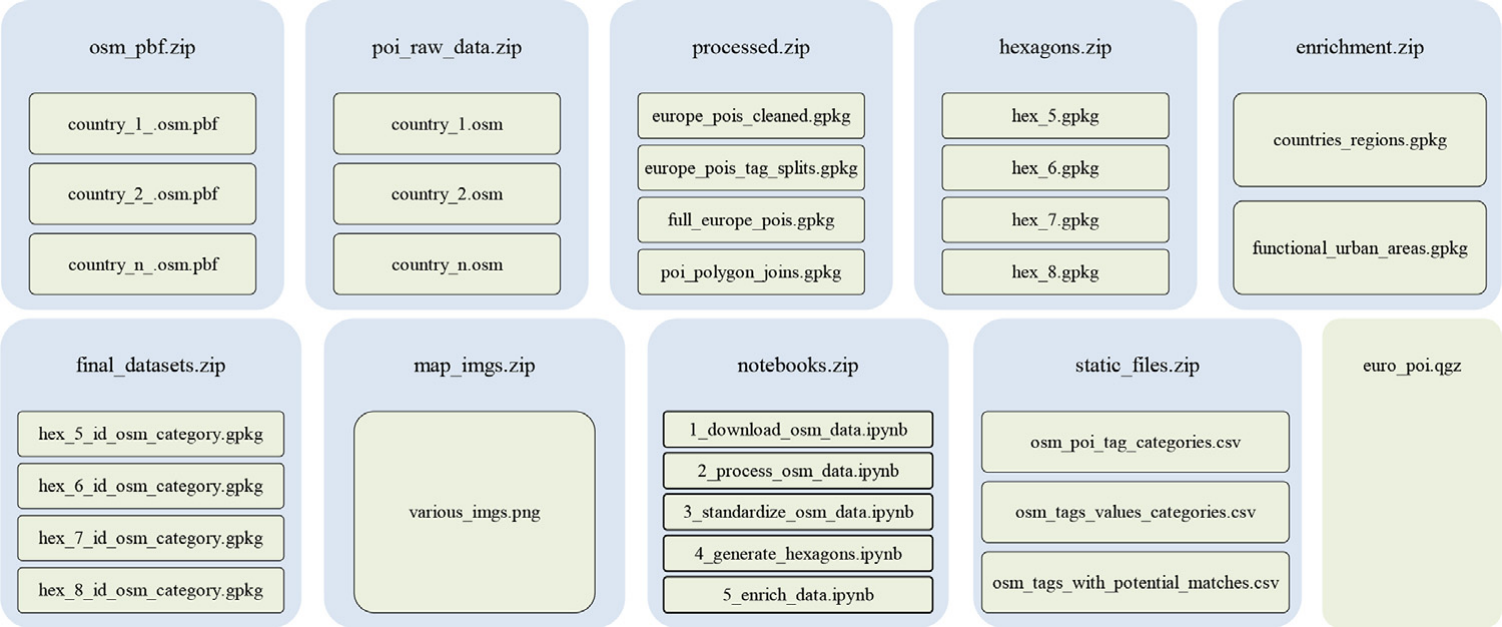
Diagram of the repository.

Our directory, outlined in Fig. 1, contains both raw and processed data as well as Python Jupyter Notebook files which we used to collect and process the data. Folders are represented in blue, and files are represented in green.

As shown in the above diagram, the file repository contains nine zip files, being osm_pbf, poi_raw_data, processed, hexagons, enrichment, final_datasets, map_imgs, notebooks, static_files, as well as an additional file named euro_poi.qgz being the associated project file for QGIS, an open-sourced spatial analysis tool. The data for this project is outlined as follows—

• **osm_pbf**, holding raw OSM data for 50 countries designated to be in Europe by GeoFabrik.
• **poi_raw_data**, holding extracted and filtered versions of each of the 50 countries in the osm_pbf folder to contain only POI data.
• **processed**, holding four files— europe_pois_tag_splits.gpgk being a dataset of all tags associated with each POI point (i.e. containing land use information, transit features, and other POI accessory data); full_europe_pois.gpkg being the full European POI dataset after the tags have been split and filtered only to retain relevant POI information; europe_pois_cleaned.gpkg being the cleaned POI data, as outlined in the methods; and lastly, poi_polygon_joins.gpkg being the euope_pois_cleaned.gpkg dataset with the relevant hexagon grid IDs.
• ***hexagons,*** holding GeoPackages for each hexagonal grid (level 5, 6, 7, and 8 resolutions),
• enrichment, which has GeoPackage (.gpkg) files containing data on country and functional urban areas that are used to enrich the final dataset further.
• ***final_datasets,*** holding the final cleaned, standardized, and enriched datasets at levels 5, 6, 7, and 8 resolution hexagonal grids showing counts of POIs by category and features to show the urban area, country, and region the hexagon is in.

The *map_imgs* folder simply holds images generated during the methods section. The *notebooks* folder, outlined more in detail in the methods section, holds the Python Jupyter Notebooks used to extract and process the aforementioned data files. Lastly, the *static_files* folder holds three files, *osm_poi_tag_categories.csv, osm_tags_values_categories.csv,* and *osm_tags_with_potential_matches.csv* all pertaining to the data standardization section of this paper and categorizing the POI tags.

The pre-hexagon-gridded POI dataset, stored in the file path ../processed/poi_polygon_joins.gpkg, is described in Table 1. This dataset does not contain the raw OSM data itself but instead includes the POI points after preliminary data cleaning, which will be outlined further in the methods section. Fig. 1 provides a spatial visualization of this dataset, which contains 7,218,304 data points. This dataset has nine columns (Table 1), being - osm_id, representing the ID for the data point designated in the raw dataset; osm_tag, being the tag given to the data point from OSM; osm_tag_standardized being the tag after standardization was applied (further discussed in the methods); osm_tag_category representing the category for the OSM tag coming from the OSM documentation; four fields (hex_5_id, hex_6_id, hex_7_id, and hex_8_id) being the ID for each of the hexagonal grids that were spatially joined to the point dataset; and, lastly, the geometry field represents the spatial geometry for each of the data points (x-, y-coordinates) allowing the data to be used in spatial analyses and software.

**Table 1:** POI data stored in poi_polygon_joins.gpkg.

Column name	Column description	Data example
osm_id	OSM ID for the point	5232347518
osm_tag	OSM tag	Café
osm_tag_standardized	Standardized OSM tag	cafe
osm_tag_category	Category for OSM tag	food_beverage
hex_5_id	ID for the associated hexagon at resolution 5	8835a93231fffff
hex_6_id	ID for the associated hexagon at resolution 6	8535a933fffffff
hex_7_id	ID for the associated hexagon at resolution 7	8635a9327ffffff
hex_8_id	ID for the associated hexagon at resolution 8	8735a9323ffffff
geometry	Spatial reference for the point	POINT (-28.82613 38.59680)

**Fig. 2:**
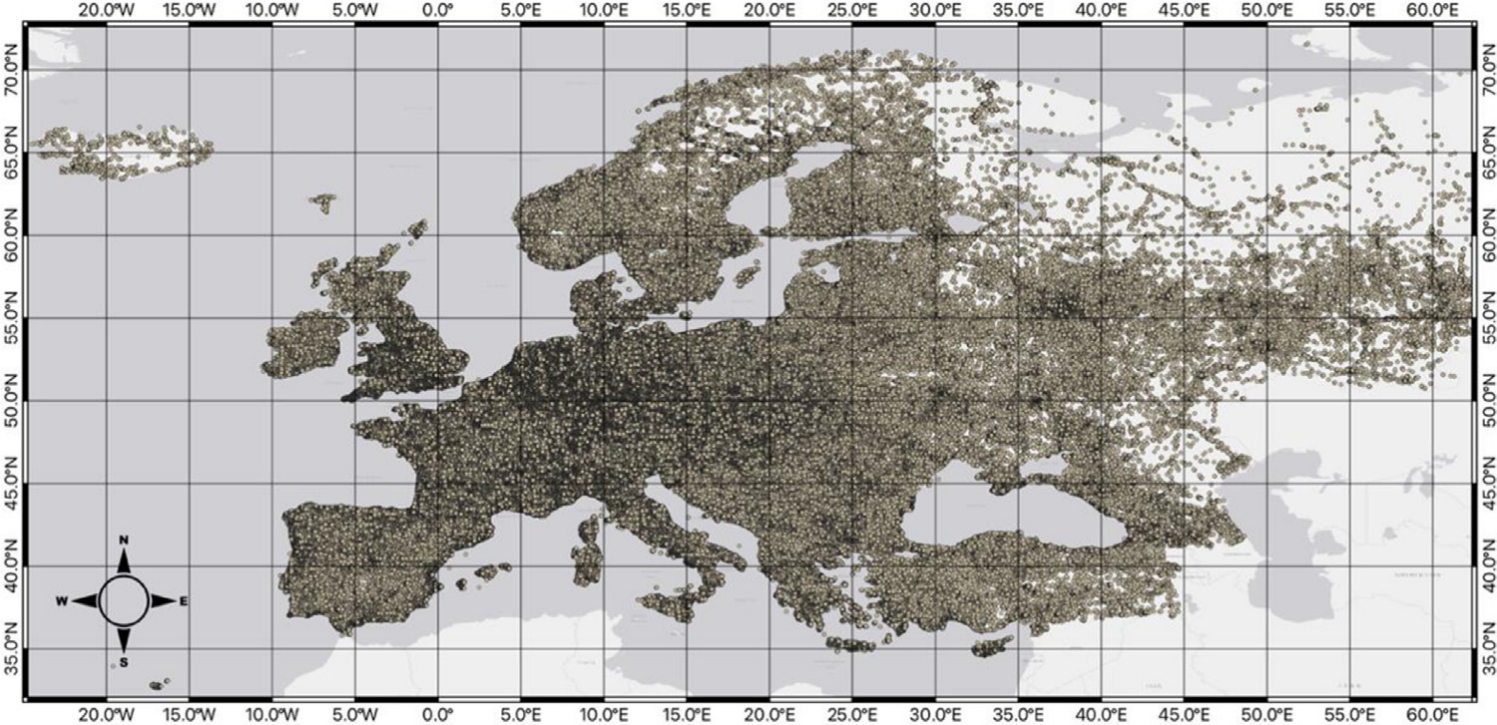
Map of the raw POI data points across the study area.

**Fig. 3:**
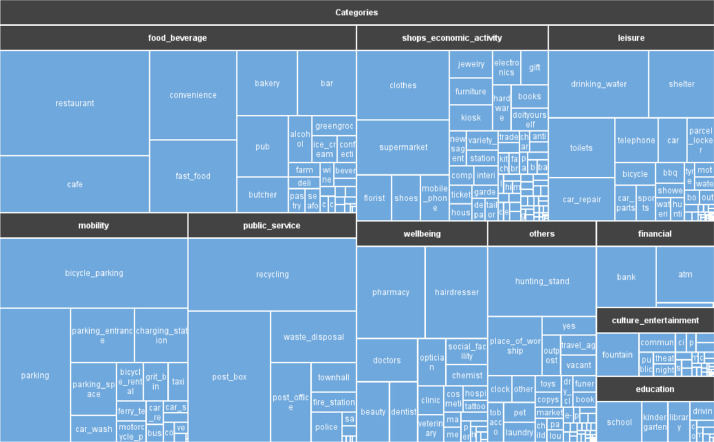
Tree plot of the categories and their associated tags.

There are multiple categories, making it challenging to describe and represent each. To aid this explanation, a tree plot was constructed (Fig. 3) to show the largest representations of OSM POI tags in each category. As well, Table 2 describes the fields of the hexagon-gridded POI dataset.

**Table 2:** Column examples in the hexagon gridded dataset (resolution 5, 6, 7, and 8).

Column name	Column description
fid	Index of the row
hex_{n}_id	Hex ID with *n* representing the resolution (5, 6, 7, 8) of the hexagon
culture_entertainment	Count of amenities related to cultural and entertainment purposes.
education	Count of educational facilities (both private and public)
financial	Count of amenities supporting financial practices (ATMs, banks, etc.)
food_beverage	Count of amenities with facilities for food and/or beverages.
leisure	Count of amenities related to parks, outdoor activities, watersports, etc.
mobility	Count of amenities related to parking, vehicle infrastructure, public transit, etc.
public_service	Count of amenities related to government-run and other public-serving facilities.
shops_economic_activity	Count of amenities related to shopping markets, malls, department stores, etc.
wellbeing	Count of amenities related to doctors, clinics, hospitals, and cosmetic facilities.
others	Count of amenities not fitting any specific category.
total_pois	The total count of POIs in an area.
functional_urban_area_name	The name of the functional urban area, if exists.
area_name	The country name of the area the hexagon primarily covers.
area_continent	The continent that the hexagon primarily covers.
area_un_region	The United Nations designated region that the hexagon primarily covers.
area_wb_region	The World Bank designated region that the hexagon primarily covers.
area_subregion	The subregion that the hexagon primarily covers.

Below, each of the hexagon grid datasets is displayed. For visualization purposes, data for each map uses the Fisher Jenks algorithm to group the *total_poi_count* field into 8 classifications.

**Fig. 4:**
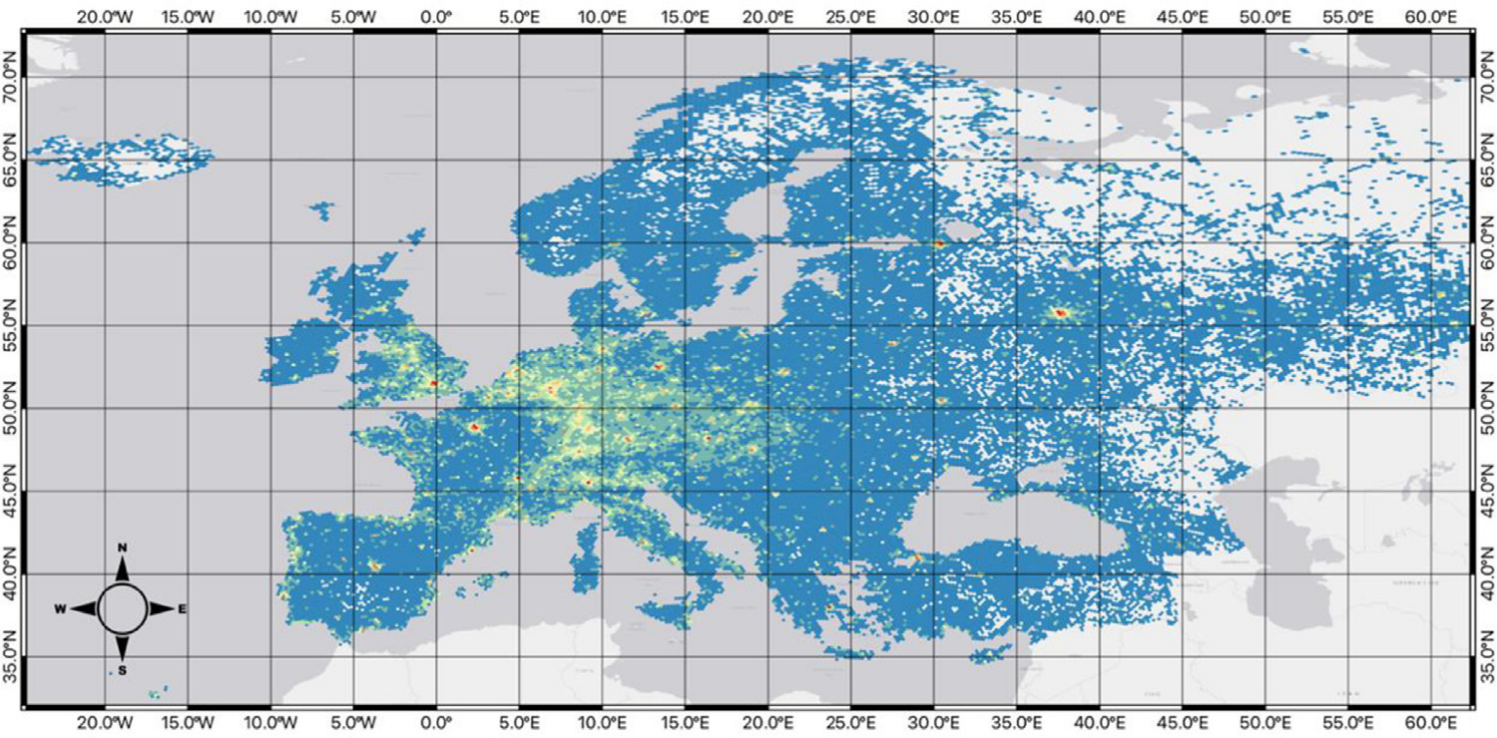
Map of the hexagon grid (resolution 5) and point counts across the study area.

Fig. 4, coming from *../final_datasets/hex_5_id_osm_category.gpkg*, represents the level-5 resolution of the hexagonal grid across the study area, along with a visualization of the number of points within each of the cells grouped by its total_poi_count using Fisher Jenks into 8 categories. There are a total of 36,890 hexagons, with each being approximately 250km^2^ large.

**Fig. 5:**
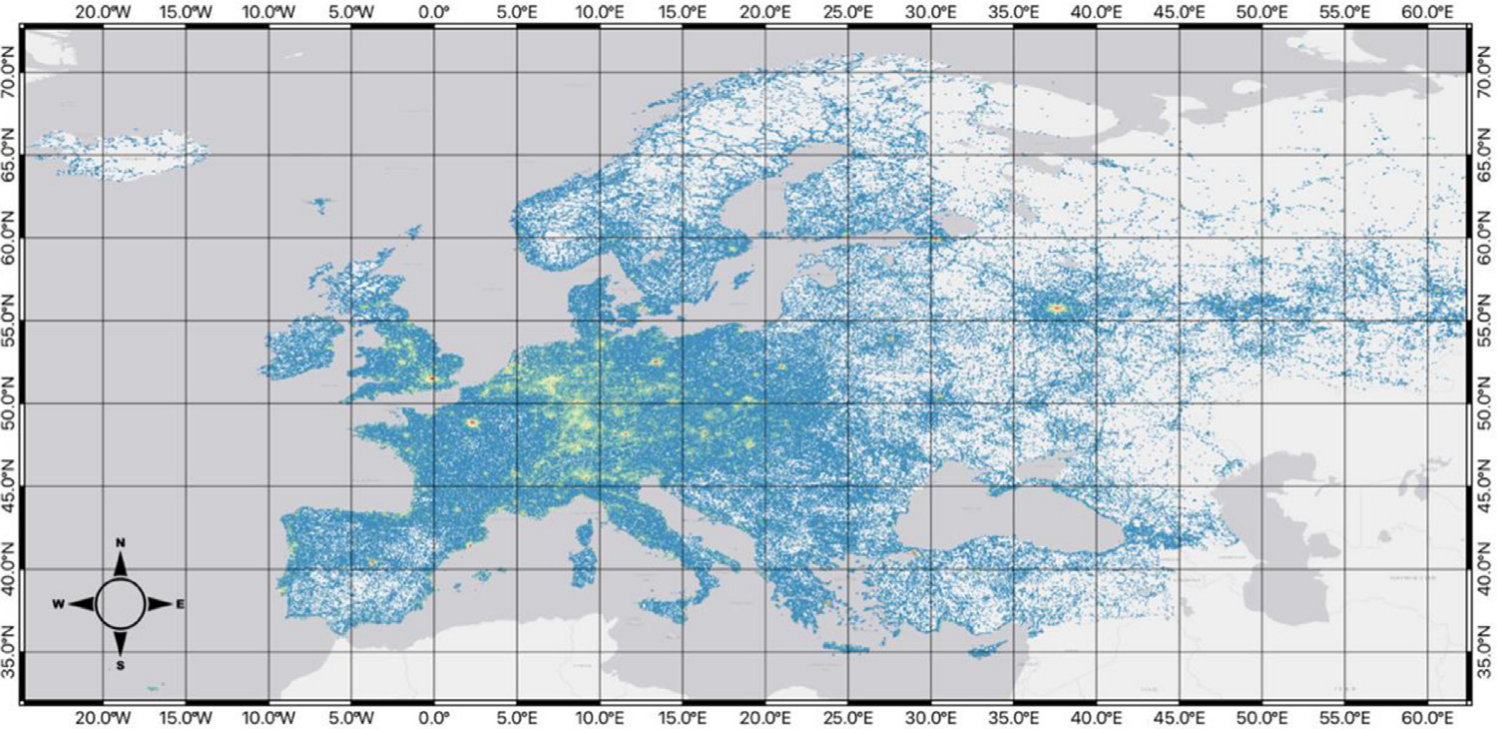
Map of the hexagon grid (resolution 6) and point counts across the study area.

Fig. 5, coming from *../final_datasets/hex_6_id_osm_category.gpkg*, shows the level-6 resolution of the hexagonal grid, with each hexagon having an approximate area of 35km^2^ across the study area with 151,662 hexagons. Similar to the level-5 resolution, a classification of the total_poi_count into 8 classes with the Fisher Jenks algorithm was used to visualize the data clearly.

**Fig. 6:**
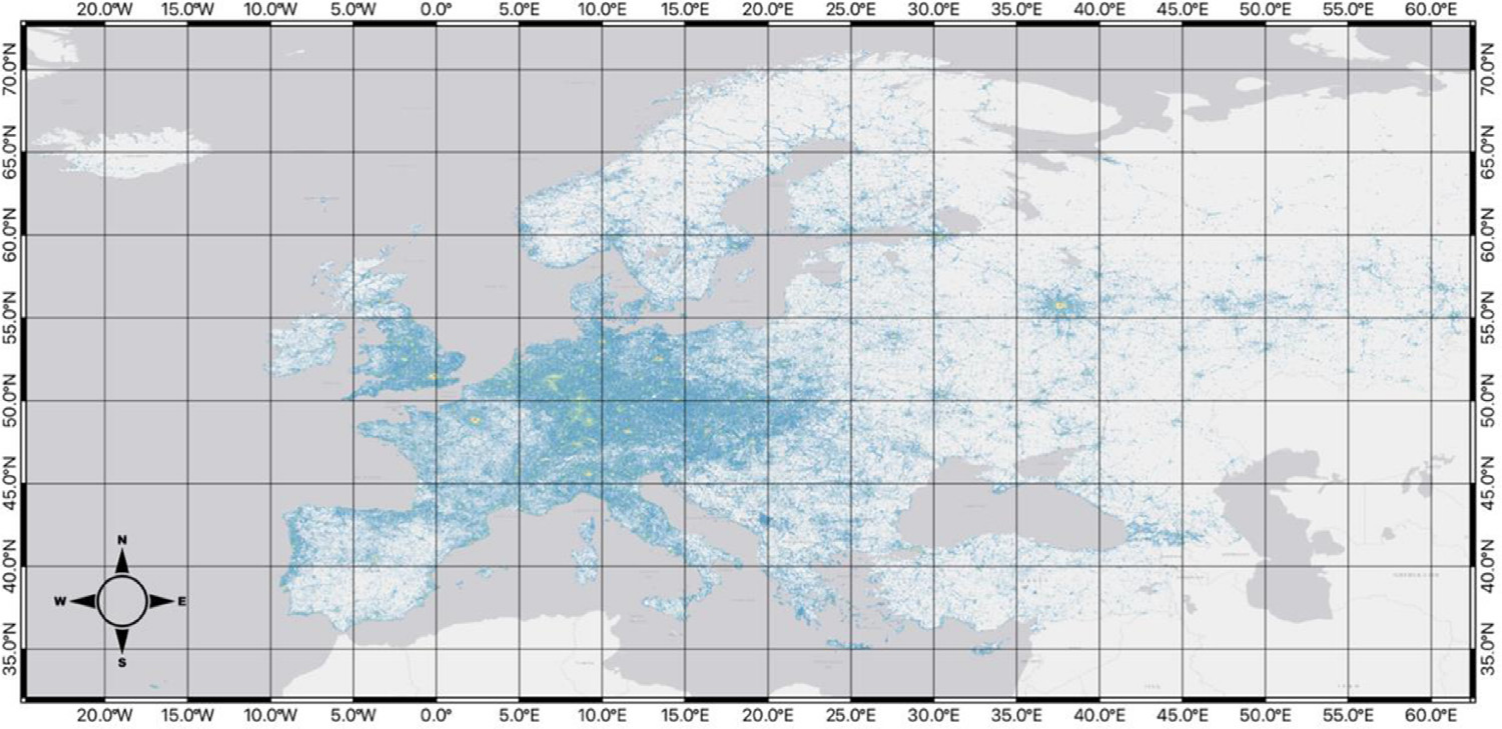
Map of the hexagon grid (resolution 7) and point counts across the study area.

Fig. 6, coming from *../final_datasets/hex_7_id_osm_category.gpkg*, signifies the level-7 resolution of the hexagonal grid, showing 451,999 hexagons across the study area, with each being approximately 5km^2^. Again, with a Fisher Jenks grouping of total_poi_count into 8 categories.

**Fig. 7:**
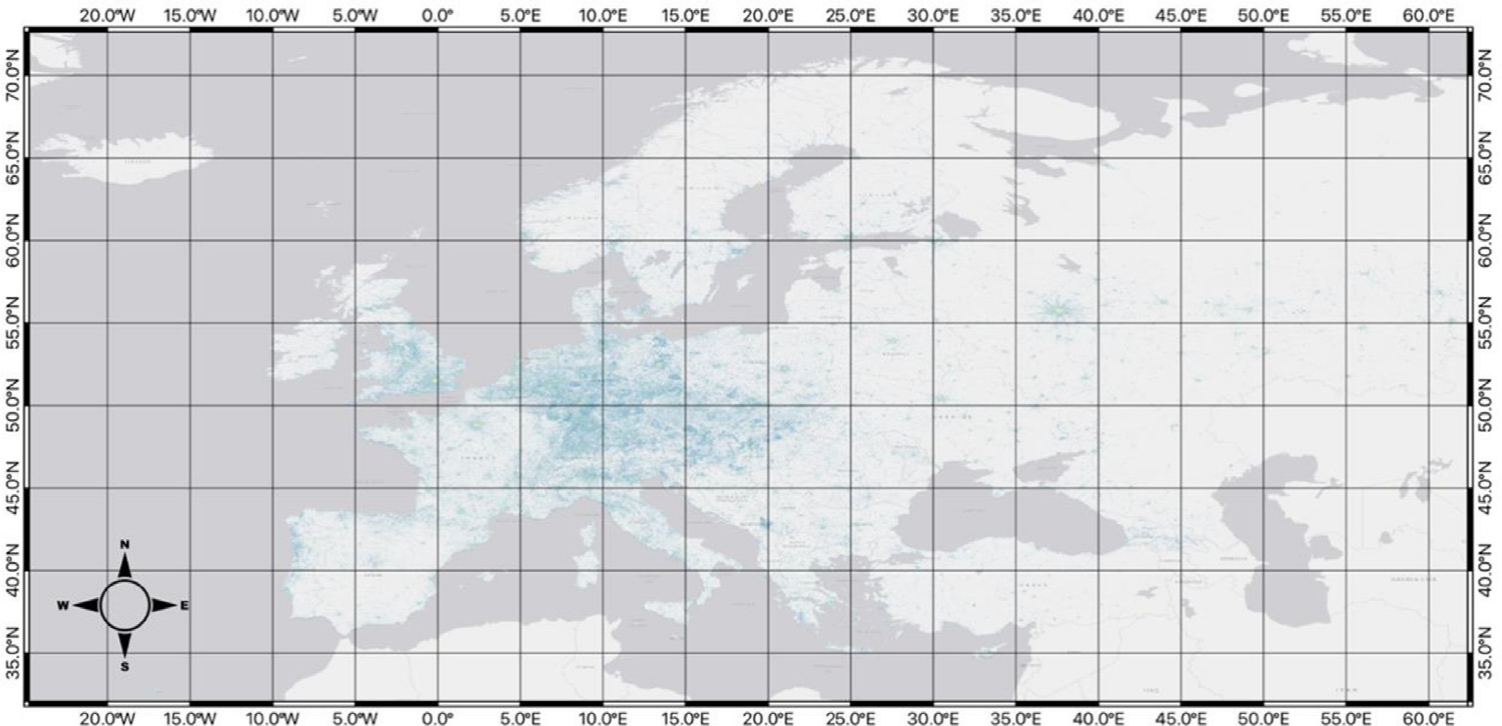
Map of the hexagon grid (resolution 8) and point counts across the study area.

Fig. 7, coming from *../final_datasets/hex_8_id_osm_category.gpkg*, represents the level-8 resolution of the hexagonal grid with 988,575 hexagons, visualized with an 8-class grouping of the total_poi_count field using the Fisher Jenks algorithm. Leve-8 is the smallest resolution size, with each hexagon having an area of approximately 0.7 km^2^.

**Fig. 8:**
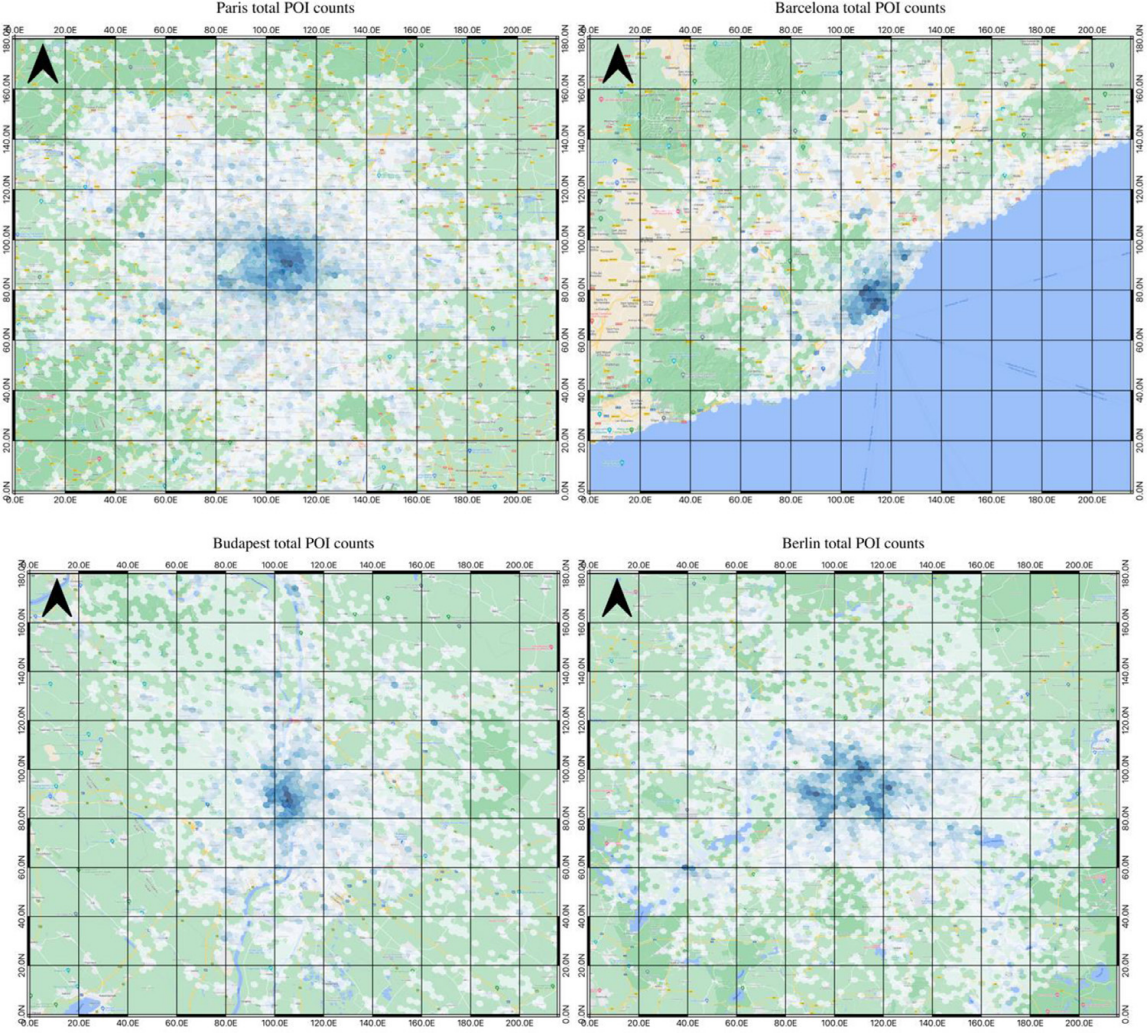
Hex-Grid POI counts for different cities (Paris, Barcelona, Berlin, Budapest).

To show the data at the city level, the total POI count data for four cities (Paris, Barcelona, Budapest, and Berlin) is displayed in Fig. 8. For each map, the hexagonal grid is visualized using the Fisher Jenks algorithm to group the counts into ten groups, with darker blue indicating higher counts of POIs and lighter blue indicating lower counts. These produced visualizations are as expected, having the highest counts of POIs in the urban centers with satellite cities and hubs being visible around their own respective POI hotspots. However, it is important to note that the purpose of this visualization is not to analyze the urban areas but to show this dataset's ability to visualize a range of areas at the same resolution, highlighting a primary benefit of using this dataset.

**Fig. 9:**
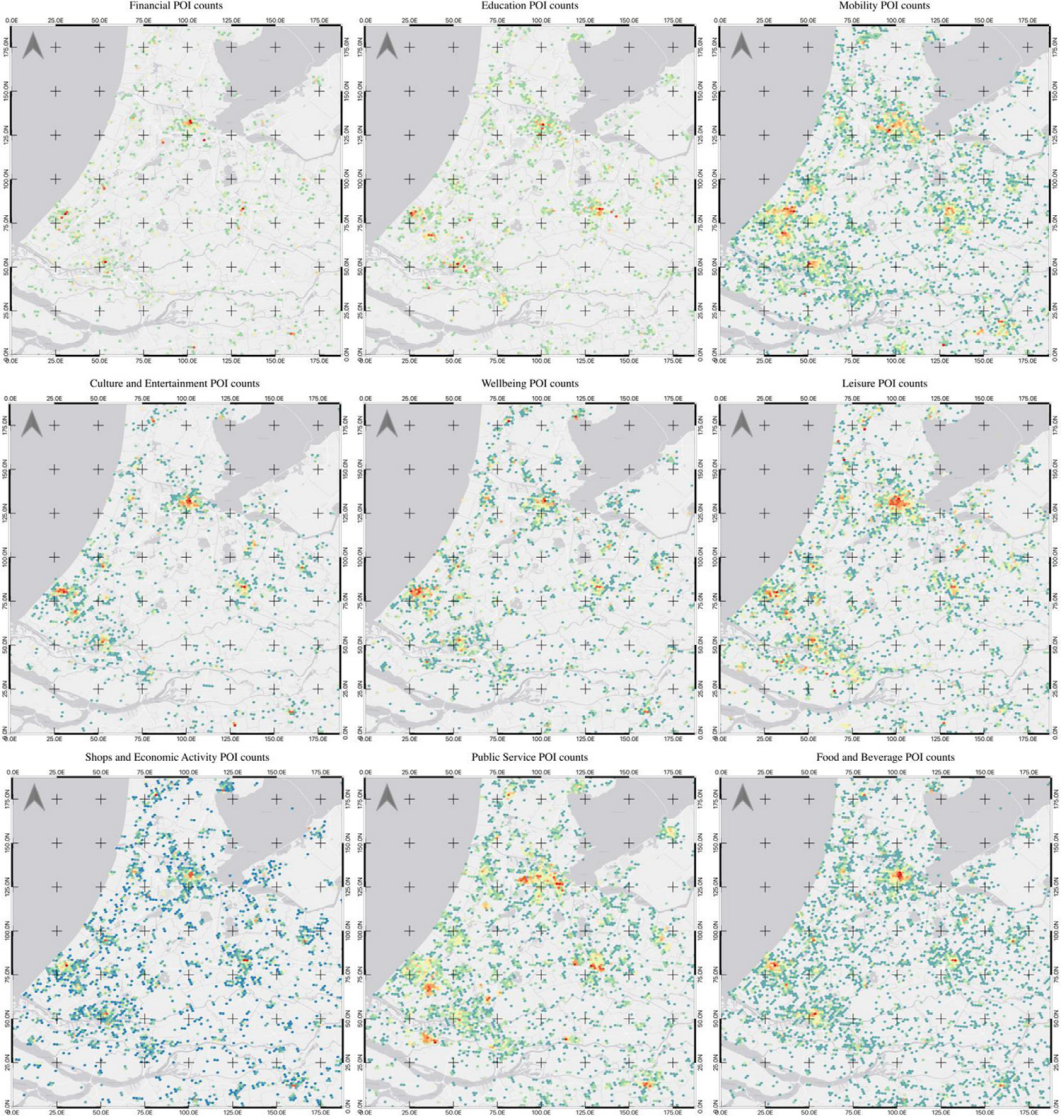
POIs by category across the western Netherlands (Rotterdam, The Hague, Utrecht, Amsterdam).

To further explain the dataset, Fig. 9 displays the different POI categories across the western side of the Netherlands (Rotterdam, The Hague, Utrecht, and Amsterdam being the main urban centers here). These maps display the same level-8 resolution hexagonal grid across this area, using the Fisher Jenks algorithm to separate the POI counts for each respective category into eight groups, with blue showing the least number of POIs, yellow showing an increased number, and red showing the hexagons with the highest count of POIs. For clear visualization, any hexagons that had no POI for a specific category were removed. As with the maps in Fig. 8, looking across multiple urban areas, these maps below are able to show the distribution of main POI categories across a large spatial area. This is not only beneficial for the purposes of urban studies, but as the dataset is standardized to the same lexicon and categorized using unified methods, the areas are more comparable than would have been otherwise. Moreover, aggregating the data in the hexagonal grids allows for more even distribution and visualization across the study area and is additionally advantageous due to its equidistant nature [3,8]. An additional benefit of this grid style is the ability to better examine the connectivity of the cells across the areas to conduct more in-depth research and analyses into the distributions of the different POI count categories [9].

## 3 Experimental Design, Materials and Methods

To provide a comprehensive overview of the materials and methods used in generating the dataset, this paper is accompanied by a code repository. The repository, as mentioned in Section 2 and in Fig. 1**,** includes the *notebooks* folder contains five separate notebooks (*1_download_osm_data.ipynb, 2_process_osm_data.ipynb, 3_standardize_osm_data.ipynb, 4_generate_hexagons.ipynb, 5_enrich_data.ipynb*). These notebooks demonstrate the complete process undertaken to generate the datasets within the repository (Fig. 2).

### 3.1 OSM Data Retrieval

The first step of this project involved acquiring POIs from the OSM project [1]. While there are several methods to accomplish this, the most efficient to download at the scale of this project is via direct download from an OSM data provider, in our case GeoFabrik. The dataset was retrieved on April 20, 2023, as OSM is continually being updated by the community, and GeoFabrik pushes changes made to the data to their servers daily, the data extracted can be as new as April 19, 2023. In the file repository, 1*_download_osm_data.ipynb* shows this process, where a list of download links to the files are compiled and then processed via Python script, taking advantage of the *ThreadPool* function of the *multiprocessing* package to speed up the process [10]. While it is possible to download a regional file as a whole, the proposed methodology is able to function even on less powerful machines as it breaks the download into chunks (according to the URL list input), posing less risk of kernel collapse.

### 3.2 OSM Data Processing

The second step of the process involved processing, filtering, and merging each .pbf file that was downloaded in *1_download_osm_data.ipynb* into a single file. Before starting this step, *osmosis* was installed locally to facilitate handling the .pbf files. As outlined in *2_filter_and_merge_osm_data.ipynb*, there are two sub-steps— First, the .pbf files are opened and filtered to extract only data with an “amenity” or “shop” key and then saved to a new directory as an .osm file. Second, the extracted and filtered .osm files are merged into one .osm file using *osmosis* [11] and a bash command.

### 3.3 OSM Data Standardization

The POI data was further processed and standardized in the next step, shown in *4_process_osm_poi_data.ipynb*. This was the bulk of the project and took place in the following steps.

First, a new dataset was created to merge the OSM ID, a dictionary of the tag data (if the key was “amenity” or “shop”), and the geometry of the POI point. This was then converted to a GeoDataFrame using the GeoPandas package in Python [12].

Second, it was necessary to standardize the tags as they may be non-standard (i.e., not aligning with tag/key names in the OSM documentation), be in another language, have spelling mistakes, or face other issues that come with community-driven projects. Using the already standardized key/tag terms from the OSM documentation, we were able to pose the issue of misaligned words as a problem the TF-IDF algorithm could help solve. Employing the *tfidf_matcher* package [13], the information from the OSM documentation was compiled into a lookup document that the POI tags could be run through, using the TF-IDF algorithm, to return potential matches. While the results were excellent, this method is not foolproof and prone to mistakes. To resolve those, the matches for the tags were exported, those with a 100% match were excluded, and the remainder were manually processed and checked to ensure higher accuracies. This approach could be further improved in the future as it is resource intensive in terms of human labor. After the tags were standardized, they were given a categorical label based on the category they were under in the OSM documentation. These categories were then further aggregated to be one of 'culture_entertainment', 'education', 'financial', 'food_beverage', 'leisure', 'mobility', 'others', 'public_service', 'shops_economic_activity', 'wellbeing'.

In the next phase, as demonstrated in *4_process_osm_poi_data.ipynb,* the POI data underwent further processing and standardization. This constituted the core of the project and unfolded in the following sequence of steps:

First, a new dataset was generated by merging the OSM ID, a dictionary containing tag data (when the key was "amenity" or "shop"), and the geometry of the POI point. Subsequently, this dataset was transformed into a GeoDataFrame using the GeoPandas package in Python [12].

Second, tags were standardized, as they may be non-standard and have issues that frequently arise from community-driven projects, such as being in another language, having spelling mistakes, or not aligning with tag/key names in the OSM documentation. To solve this problem, this study used already standardized key/tag terms from the OSM documentation and treated the issue of misaligned words as a problem that the TF-IDF algorithm could help solve. The tfidf_matcher package [6] was used to compile information from the OSM documentation into a lookup document. Then, we ran the POI tags through the TF-IDF algorithm to return potential matches. Although the results were promising, this method is not foolproof and may produce mistakes. To ensure higher accuracies, this study exported the tag matches, excluding those with a 100% match, and manually processed the remaining tags.

This approach can be improved in the future as it requires significant human labor. After standardizing the tags, we applied categorical labels based on the categories they belonged to in the OSM documentation. These categories were further aggregated into the following ten categories: culture_entertainment, education, financial, food_beverage, leisure, mobility, others, public_service, shops_economic_activity, and well-being.

### 3.4 Hexagonal Grid Generation

The final step of the project, detailed in *3_get_hex_grid.ipynb*, involves extracting the H3 hexagonal grid [7] using the *h3fy* function from the *tobler* package [8]. This function requires a boundary file (in a spatial format, such as .shp, .gpkg, etc.) to set the bounds for the hexagonal grid generation and an integer to indicate the desired grid resolution. This project used the POI data file generated in previous steps to create a boundary file for grid generation. For the purposes of this research, hexagonal grids were generated at the 5-, 6-, 7-, and 8-level resolutions, which correspond to approximately 250km², 35km², 5km², and 0.7km², respectively.

Next, the standardized and categorized OSM data was joined to the hexagonal grids to retrieve their respective Hex IDs. With these new ID columns, it was possible to group the individual datasets by ID and category to retrieve a count of POIs. This new dataset was then pivoted to provide the index as the hex ID, the columns as the categories, and the values as the count of POIs per category. Additionally, a "total_pois" field was created, which represents a count of all POIs in the respective hexagon.

These new datasets were joined to the hexagonal grid to create the final spatial datasets, saved in the *../final_datasets* folder. To further clean the data and save on file storage space, hexagons with 0 or NULL POI counts were dropped from the final datasets. It is worth noting that while this study chose to use the count of POI points as our aggregation statistics, it is additionally possible to calculate the density of points within a hexagon, the proportion of points in a specific category to the total POI points, or other statistics. Different aggregation statistics can provide new and different insights into the dataset; however, they are out of the scope of this current research.

### 3.5 Data Enrichment

A mentioned benefit of this dataset is its ability to be enriched with new features. To demonstrate this, *5_enrich_data.ipynb* enriches the data by adding the country name, United Nations region, World Bank region, subregion, and functional urban area into the dataset based on the hexagonal grid. This procedure was done by collecting data on countries and their regions from the Natural Earth 1:10m Cultural Vectors dataset [14] and the functional urban area data from the European Commission Global Human Settlement Layer dataset [15]. These datasets were then spatially joined to each of the hexagonal grid datasets to add in the additional features, with the join accepting only the area with the largest overlap with the hexagon. This final enrichment is beneficial as it allows researchers the ability to extract hexagons based on location or urban area but also allow for further enrichment based on the features joined in.

### 3.6 Dataset Limitations

While the OSM project is impressive and continually growing, it still holds multiple limitations due to variations in quality and completeness between cities, countries, and regions. This work has attempted to correct for many of these variations by standardizing the POI data points to the categories and labels mentioned in the OSM documentation; however, there are many issues that are not possible to correct due to a range of externalities from cultural differences to language barriers to community participation and would need further evaluation for future studies.

## Ethics Statements

This work meets the publisher's ethical requirements and does not involve studies with animals and humans. The dataset has been downloaded with permissions under the Open Database License 1.0 license.

## CRediT authorship contribution statement

**Dakota Aaron McCarty:** Conceptualization, Methodology, Writing – original draft. **Hyun Woo Kim:** Supervision, Writing – review & editing.

## Declaration of Competing Interest

The authors declare that they have no known competing financial interests or personal relationships that could have appeared to influence the work reported in this paper.

## Data Availability

A Standardized European Hexagon Gridded Dataset Based on OpenStreetMap POIs (Reference data) (Zenodo).
